# Supplementary material for: An introduction to thermodynamic integration and application to dynamic causal models
Source: Cogn Neurodyn. 2021 Jul 25;16(1):1–15. doi: 10.1007/s11571-021-09696-9 (PMC8807794; doi:10.1007/s11571-021-09696-9)
Supplement: Supplementary file 1 — Supplementary file1 (PDF 1151 KB) [file 11571_2021_9696_MOESM1_ESM.pdf]

An introduction to thermodynamic integration and application to dynamic causal models – Supplementary material

S1 A primer on Dynamic Causal Models

In this section, we provide a short introduction to dynamic causal modelling (DCM). Since the examples in the main text focus on fMRI data, and we limit our discussion to DCM for fMRI (Friston, Harrison, & Penny, 2003; K. E. Stephan et al., 2008; K. E. Stephan, Weiskopf, Drysdale, Robinson, & Friston, 2007).

DCM for fMRI is characterized by two layers: first, a set of ordinary differential equations that model the dynamics of interacting neuronal states x and local hemodynamic states h. Second, the hemodynamic states enter a static nonlinear observation equation that relates venous blood volume and deoxyhemoglobin content to measured BOLD signal changes. In the following, we discuss only the most relevant equations, in order to convey an understanding of the type of problem that model inversion in DCM faces.

The general form of the dynamics of the neuronal layer is

$$\frac{dx}{dt} = f(x, u, \theta_c) \tag{1}$$

where $x = (x_1, \dots, x_N)$ describes the neuronal states of N regions, $u = (u_1, \dots, u_M)$ represents the time series of M experimental manipulations or inputs, and θ_c are the connectivity parameters that determine the neuronal dynamics. Using a second order Taylor expansion (Stephan et al., 2008), the dynamics f can be approximated as:

$$\frac{dx}{dt} = Ax + \sum_{j=1}^{\mathrm{M}} u_j B_j x + Cu + \sum_{i=1}^{\mathrm{N}} x_i D_i x\,. \tag{2}$$

The connectivity parameters θ_c can be divided into four subsets: The $N \times N$ matrix A describes endogenous connectivity strengths between regions. The set of $N \times N$ matrices $B = \{B_1, \dots, B_M\}$ encodes modulatory effects of inputs on connections between regions. The $N \times M$ matrix C describes the direct effects of driving inputs on regions. Finally, the $N \times N$ matrices $D = \{D_1, \dots, D_N\}$ denote second-order interactions between two regions

that affect a third one. Linear DCMs use A and C matrices, bilinear DCMs contain at least one non-zero B matrix, and nonlinear DCMs contain at least one non-zero D matrix. Together $\theta_c = \{A, B, C, D\}$ fully describe the dynamics of the neuronal layer.

The hemodynamic model of DCM originates from the Balloon model proposed by Buxton, Wong, and Frank (1998) and extended by Friston (2002) and K. E. Stephan et al. (2007). In brief, it describes how changes in neuronal states locally alter cerebral blood flow, which, in turn, affects venous blood volume and deoxyhemoglobin content. The model consists of a cascade of deterministic differential equations:

$$\frac{dh}{dt} = l(h, x, \theta_h), \quad (3)$$

where $h = (h_1, \ldots, h_N)$ denotes hemodynamic states in each of N regions. Detailed equations and the meaning of the hemodynamic parameters θ_h can be found in K. E. Stephan et al. (2007). It is worth noting that the hemodynamic equations are nonlinear and that the original implementation in SPM uses a local (bi)linear approximation (Friston et al., 2003).

Finally, hemodynamic states enter a static nonlinear observation equation g with parameters θ_g that models the BOLD signal y:

$$y = g(h, \theta_g) + X_0\beta + \varepsilon \quad (4)$$

The term X_0 is a matrix of confound regressors that accounts for constant terms and low frequency fluctuations. The Gaussian observation noise ε is characterized by the covariance matrix Π_ϵ^{-1}:

$$\varepsilon \sim N(0, \Pi_\epsilon^{-1}). \quad (5)$$

The precision matrix Π_ϵ is represented as a linear combination $\Pi_\epsilon = \sum_r \exp(\lambda_r)\, Q_r$. The precision components Q_r serve to account for temporal autocorrelation and regional differences in noise variance (Friston et al., 2003). Here, we assume that the time series have been whitened and therefore only account for region-specific variances. In this case, each Q_r is a diagonal matrix with diagonal elements belonging to region r set to 1, and 0 elsewhere.

To complete the generative model, the prior distribution of the parameters $\Theta = (\theta_c, \theta_h, \theta_g, \beta)$ and hyperparameters Λ needs to be specified. For the results presented in this paper, the priors have been largely matched to SPM8 release 5236

(http://www.fil.ion.ucl.ac.uk/spm), except for the scaling of the prior variance of the coefficients of the confound matrix X_0, which was adapted to the scaling of the data as explained in S8. All parameters' prior distributions are Gaussian, and when positivity needs to be enforced, an adequate transformation function is used.

S2 Bayesian model comparison and selection

In this section, we provide a summary of Bayesian model selection (BMS). Detailed treatments can be found in standard textbooks, such as MacKay (2004).

Bayesian inference involves the specification of a probabilistic or generative model m with data y and parameters θ. The model has two components: the prior density over θ, $p(\theta|m)$, and the likelihood function $p(y|\theta, m)$. These are combined to form the posterior distribution using Bayes' theorem. Conditioning on a given model m, the posterior distribution is:

$$p(\theta|y,m) = \frac{p(y|\theta,m)p(\theta|m)}{p(y|m)}, \quad (6)$$

$$p(y|m) = \int p(y|\theta,m)p(\theta|m)d\theta. \quad (7)$$

The normalization constant in the denominator, $p(y|m)$, is known as the marginal likelihood or model evidence and corresponds to the likelihood of the data after marginalizing out the parameters of the model.

In practice, given the monotonicity of the logarithmic function, either the evidence or its logarithm can be used to score a set of candidate models $m_1, \ldots, m_n$ (Bayesian model comparison) and to identify the best model within the model space studied (Bayesian model selection; BMS). One common metric for assessing the relative goodness of two models is the Bayes factor (Kass & Raftery, 1995):

$$B_{i,j} = \frac{p(\,y \mid m_i\,)}{p(\,y \mid m_j\,)}. \quad (8)$$

or, equivalently, the exponential of the difference in LME of two models.

BMS has gained an important role in neuroimaging, not only for DCM but also in other contexts requiring model comparison, such as EEG source reconstruction (Henson, Mattout, Phillips, & Friston, 2009; Wipf & Nagarajan, 2009), or computational

neuroimaging (Friston & Dolan, 2010; Klaas E. Stephan, Iglesias, Heinzle, & Diaconescu, 2015; K. E. Stephan et al., 2017). Group-level BMS techniques exist which account for individual heterogeneity by treating the model as a random variable in the population (Friston et al., 2016; Rigoux, Stephan, Friston, & Daunizeau, 2014; K. E. Stephan, Penny, Daunizeau, Moran, & Friston, 2009). Finally, Bayesian model averaging allows one to compute an average posterior over models (Penny et al., 2010; Trujillo-Barreto, Aubert-Vázquez, & Valdés-Sosa, 2004), weighted by the posterior probability of each model. Critically, these approaches rely on an accurate estimate of each model's evidence.

As mentioned above, except for some special cases, the model evidence cannot be determined analytically, and one typically has to resort to approximations. One computationally efficient option is VB {for textbook treatments, see \Koller, 2009 #413;MacKay, 2004 #35}, which provides a lower bound of the LME. An alternative, which we explore in detail here, is MCMC sampling. This family of methods is characterized by simulating a Markov process whose stationary distribution corresponds to the posterior distribution $p(\theta|y,m)$ (for a textbook reference, see Robert & Casella, 2010).

S3 A primer on Markov chain Monte Carlo

In this section, we provide a short introduction to Markov chain Monte Carlo (MCMC). Thermodynamic integration (TI) requires obtaining samples from a series of power posterior distributions $p_i(\theta|y,m) \propto p(y|\theta,m)^{\beta_i} p(\theta|m)$, with $0 = \beta_0 < \beta_1 \dots < \beta_N = 1$. An efficient way to achieve this is to use independent Markov chain Monte Carlo (MCMC) samplers (one for each of the β_i) to generate samples from the power posteriors.

MCMC is a powerful technique that can be used to generate samples from any arbitrary target probability distribution $p(x)$, as long as $p(x)$ can be evaluated for any given argument x, up to a multiplicative constant c. c can be unknown, but has to be constant, i.e. cannot depend on x. To this end, the MCMC sampler generates a chain of samples where each sample depends on the previous sample in the chain, but collectively, the set of all samples in the chain are distributed according to the target distribution $p(x)$. To guarantee the latter point, the samples in the chain are generated sequentially according to the following procedure: Let x_t be the last sample currently in the chain, generate a so-called proposal x' via a proposal distribution $q(x'|x_t)$. The simplest way to do this is by

adding zero-mean Gaussian noise to x_t. Then calculate the so-called Metropolis-Hastings acceptance rate a, given by:

$$a = \min\left(1, \frac{p(x')q(x\_t|x')}{p(x_t)q(x'|x_t)}\right).$$

Finally, draw a random number u that is uniformly distributed between 0 and 1. If $u < a$, the proposal is accepted and appended to the end of the chain ($x_{t+1} = x'$), otherwise the proposal is rejected and the last sample is repeated ($x_{t+1} = x_t$).

Following these steps, it is guaranteed that in the limit of an infinitely long chain, the elements of the chain represent samples from the target distribution, irrespective of the value of the first sample in the chain. More detailed treatments of MCMC can be found in standard textbooks (Brooks, Gelman, Jones, & Meng, 2011). In practice, the fact that MCMC algorithm can only run for a finite time needs to be taken into account. In this context, it is necessary to (1) account for the starting position of the chain and (2) monitor the convergence of the algorithm, i.e. to determine if the MCMC algorithm has already run for long enough such that the elements of the chain can be regarded as approximately representing samples from the desired target distribution.

The first problem is typically dealt with by discarding a number of samples at the beginning of the chain (typically the first half). The discarded part of the chain is generally referred to as burn-in period.

For the second problem, several techniques have been developed to assess the convergence of a MCMC sampler. One popular method, which is used throughout this paper, is the Gelman-Rubin's potential scale reduction factor $\hat{R}$ (Gelman & Rubin, 1992). This method tests parameter-wise convergence by comparing the variance of segments of the chains. A $\hat{R}$ statistic below 1.1 is a commonly accepted criterion for convergence. To compute this score, the samples of the log likelihood of the first (after the burn-in phase) and last third section of each chain were compared.

Since TI already requires obtaining samples from a series of power posterior distributions, convergence of the MCMC samplers can be expedited by adopting a population MCMC approach in which chains associated with neighboring temperatures (i.e., β_i and β_{i+1}) are allowed to interact by means of a "swap" accept-reject (AR) step (McDowell, Dyckman, Austin, & Clementz, 2008; Swendsen & Wang, 1986). In brief, population MCMC defines a joint product distribution

$$\prod_{i=0}^{N} p(\theta_i|y,\beta_i,m) = \prod_{i=0}^{N} \frac{p(y|\theta_i,m)^{\beta_i} p(\theta_i|m)}{Z_i}, \quad (9)$$

where N is the number of distributions or chains. The goal is to obtain samples from this distribution by two types of AR steps: First, local steps are used to sample parameters θ_i from $p_{\beta_i}(\theta_i|y,m)$. Second, samples are obtained using the swapping step in which a set of neighboring parameters θ_i, θ_{i+1} are randomly chosen and then exchanged between chains with probability:

$$\min(1, (p(y|\theta_{i+1},m)^{\beta_i} p(\theta_{i+1}|m)/((p(y|\theta_i,m)^{\beta_{i+1}}\, p(\theta_i|m))). \quad (10)$$

This AR step does not change the stationary distribution of any of the chains.

Population MCMC can be easily parallelized, with or without exploiting GPUs (Aponte et al., 2016) as each of the chains is independent of the rest of the ensemble. Swapping steps need to be performed serially but, assuming that the likelihood and prior functions have been already evaluated, this method increases the efficiency of the sampling scheme while only inducing negligible computational costs (for example, Aponte et al., 2016; Calderhead & Girolami, 2009). Intuitively, the increase in efficiency is achieved by exploring the sampling space in a way comparable to simulated annealing, i.e., allowing some of the chains to explore the parameter space more freely by relaxing the likelihood function.

S4 Derivation of the equilibrium distribution for the ideal gas example

In this section, we present the derivation of the equilibrium distribution for the ideal gas example in the main text. As outlined in the main text, the equilibrium distribution is attained as the maximum entropy solution, which can be found using a variational Lagrangian with two constraints represented by the Lagrange multipliers λ_1 and λ_2 (see Blundell & Blundell, 2009; Jaynes, 1957):

$$\frac{\delta}{\delta q}\left[-k_B \int q(\theta) \ln q(\theta)\, d\theta - \lambda_1 \left(\int q(\theta)\phi(\theta) d\theta - U\right) - \lambda_2 \left(\int q(\theta) d\theta - 1\right)\right] = 0. \quad (11)$$

Noting that

$$-\frac{\delta}{\delta q} k_B \int q(\theta) \ln q(\theta)\, d\theta = k_B(-1 - \ln q(\theta)) \quad (12)$$

$$-\frac{\delta}{\delta q}\lambda_1\left(\int q(\theta)\phi(\theta)d\theta - U\right) = -\lambda_1\phi(\theta), \tag{13}$$

$$-\frac{\delta}{\delta q}\lambda_2\left(\int q(\theta)d\theta - 1\right) = -\lambda_2, \tag{14}$$

the Lagrangian yields

$$k_B \ln q(\theta) = -\lambda_1\phi(\theta) - \lambda_2 - k_B, \tag{15}$$

$$q(\theta) = \frac{1}{\exp\left(\frac{\lambda_2}{k_B} + 1\right)}\exp\left(-\frac{\lambda_1}{k_B}\phi(\theta)\right). \tag{16}$$

The term λ_1 constitutes the definition of inverse temperature in statistical physics (Blundell & Blundell, 2009; Jaynes, 1957):

$$\frac{1}{T} \stackrel{\text{def}}{=} \lambda_1. \tag{17}$$

The term $\frac{\lambda_1}{k_B} = \frac{1}{k_B T}$ is commonly represented by the symbol β. In order to derive the second constant λ_2, we write:

$$q(\theta) = \frac{1}{Z}\exp\left(-\frac{\phi(\theta)}{k_B T}\right), \tag{18}$$

where Z is referred to as the partition function of the system:

$$Z \stackrel{\text{def}}{=} \int \exp\left(-\frac{\phi(\theta)}{k_B T}\right) d\theta\,. \tag{19}$$

Hence, the term $\exp\left(\frac{\lambda_2}{k_B} + 1\right)$ is the normalization constant of $q(\theta)$, and thus $\lambda_2 = k_B(\ln Z - 1)$.

S5 Variational Bayes under the Laplace approximation for DCM

This section introduces the variational Bayes under the Laplace (VBL) approximation for inverting dynamic causal models. For an in-depth discussion see (Friston, Mattout, Trujillo-Barreto, Ashburner, & Penny, 2007).

Commonly, in order to maximize $-F_{VB}$, a mean field approximation of q is used. In other words, the distribution q is assumed to factorize into different sets of parameters, each of which defines a more tractable optimization problem. In the case of DCM, q is assumed to have the form:

$$q(\Theta, \mathit{\Lambda}) = q(\Theta)q(\Lambda), \tag{20}$$

i.e., the parameters $\Theta = (\theta_c, \theta_h, \theta_g, \beta)$ and the hyperparameters Λ are assumed to be conditionally independent. The functional $-F_{VB}$ can be optimized iteratively with respect to Θ and Λ converging to a maximum $-F_{VB} \leq \ln p(y|m)$ (Koller, 2009). This rests on maximizing the variational energies:

$$\ln q(\mathit{\Theta}) = \int q(\mathit{\Lambda}) \ln p(y, \mathit{\Theta}, \mathit{\Lambda}) d\mathit{\Lambda} + c_{\mathit{\Theta}}, \tag{21}$$

$$\ln q(\mathit{\Lambda}) = \int q(\mathit{\Theta}) \ln p(y, \mathit{\Theta}, \mathit{\Lambda}) d\mathit{\Theta} + c_{\mathit{\Lambda}}. \tag{22}$$

where $c_{\mathit{\Theta}}$ and $c_{\mathit{\Lambda}}$ are constants with respect to $\mathit{\Theta}$ and $\mathit{\Lambda}$, respectively. In DCM, it is typically assumed that all terms are Gaussian (but see Raman, Deserno, Schlagenhauf, and Stephan (2016) and Yao et al. (2018) who used conjugate priors for the noise terms).

Despite the mean field approximation, the integrals in Eq. 22 and 21 and cannot be solved analytically because of the nonlinearities of the forward model (Eq. 4). This problem is circumvented by approximating the log of the unnormalized posterior with a second order Taylor expansion on a local maximum (or equivalently, the unnormalized posterior is assumed to be Gaussian) and optimizing the objective function $\ln p(y, \mathit{\Theta}, \mathit{\Lambda})$ through gradient ascent (but see Lomakina et al. (2015) for an alternative based on Gaussian processes). This approach is called the Laplace approximation (Friston et al., 2007) and underlies other methods such as BIC (Schwarz, 1978) or when the normalization constant of an approximate, tractable posterior is directly used (Kass & Raftery, 1995). As a consequence of this approximation, the variational free energy is no longer guaranteed to represent a lower bound on the log evidence (Wipf & Nagarajan, 2009). **A detailed treatment of VBL can be found in Friston et al. (2007). In section S9, we present a simplified version of the derivation of the VBL estimate of the free energy and an explicit expression for the accuracy term.**

The VBL algorithm used here was the implementation available in the software package SPM8 (release 5236), which employs a gradient ascent scheme to optimize the marginal distributions $q(\Theta)$ and $q(\Lambda)$ (Friston et al., 2007).

S6 Conventional sampling-based estimation of model evidence

In this section, we provide summaries to two popular sampling-based estimators for the log model evidence: the prior arithmetic mean estimator (AME) and the posterior harmonic mean estimator (HME).

Prior arithmetic mean estimator (AME)

Importance sampling is a Monte Carlo method for approximating the expected value of a random variable *h(X)* under the density p by means of an auxiliary density function $w(X)$, which is required to be absolutely continuous with respect to p (Robert & Casella, 2010; p. 92, Def. 3.9), or less formally, the auxiliary density w should share the same support as p to avoid zeros in the denominator:

$$\int h(x)p(x)dx = \int \frac{h(x)p(x)w(x)}{w(x)}dx. \tag{23}$$

From the strong law of large numbers, if this expected value exists, the process

$$\lim_{\mathrm{K}\to\infty} \frac{1}{\mathrm{K}} \sum_{k=1}^{K} h(x_i) \frac{p(x_k)}{w(x_k)} \tag{24}$$

converges almost surely to Eq. 9 when the samples $x_1, \dots, x_K$ have been drawn from the auxiliary distribution w.

In order to approximate the model evidence by importance sampling, the simplest choice of the auxiliary density is the prior distribution, $w = p(\theta \mid m)$. This results in the prior arithmetic mean estimator (AME):

$$p(y|m) = \int p(y|\theta, m)p(\theta|m)d\theta = \int p(y|\theta, m)p(\theta|m)\frac{p(\theta|m)}{p(\theta|m)}d\theta\,, \tag{25}$$

$$p_{AME} = \frac{1}{K}\sum_{k=1}^{K} p(y|\theta_k, m)\,. \tag{26}$$

where samples θ_k have been obtained from the prior distribution $p(\theta|m)$. Because samples of the likelihood $p(y|\theta, m)$ can greatly exceed the range of double precision

floating point numbers, it is necessary to normalize the likelihood function in log space. This can be achieved with the following formula:

$$\ln p_{AME} = \ln \alpha - \ln K + \ln \sum_{i=1}^{K} \exp[\ln p(y|\theta_i, m) - \ln \alpha], \tag{27}$$

where $\alpha > 0$ is an arbitrary constant. In all analyses reported here, α was set to $\max_k p(y|\theta_k, m)$.

A serious shortcoming of AME is that in the great majority of situations most samples drawn from the prior have very low likelihood. Therefore, an extremely large number of samples is required to ensure that high likelihood regions of the parameter space are taken into account by the estimator; otherwise, the estimator suffers from high variance (Vyshemirsky & Girolami, 2008).

Posterior harmonic mean estimator (HME)

The second choice for the auxiliary density is the posterior distribution, which results in the posterior harmonic mean estimator (HME). This estimator has received divergent appraisals in the literature as a method for computing the LME (for example, Kass & Raftery, 1995; Wolpert & Schmidler, 2012). Re-expressing the model evidence, the HME can be derived as follows:

$$\begin{aligned} \frac{1}{p(y|m)} &= \int \frac{p(\theta|m)}{p(y|m)} d\theta, \\ &= \int \frac{p(y|\theta, m) p(\theta|m)}{p(y|\theta, m) p(y|m)} d\theta, \\ &= \int \frac{p(\theta|y, m)}{p(y|\theta, m)} d\theta \end{aligned} \tag{28}$$

$$p_{HME} = \left(\frac{1}{K} \sum_{i=1}^{K} \frac{1}{p(y|\theta_i, m)} \right)^{-1}. \tag{29}$$

Here, samples θ_i are drawn from the posterior distribution $p(\theta|y, m)$.

In order to avoid numerical instabilities, it is again necessary to normalize in log space, using the formula

$$\ln p_{HME} = \ln K + \ln \alpha - \ln \sum_{i=1}^{K} \exp[-\ln p(y|\theta_{\mathrm{i}}, m) + \ln \alpha]. \tag{30}$$

Here, $\ln \alpha$ has been chosen to be $\max_i -\ln p(y|\theta_i, m)$.

A disadvantage of HME is that its variance might be infinite when the likelihood function is not heavy-tailed (Raftery, Newton, Satagopan, & Krivitsky, 2007), which has serious consequences for the convergence rate of a wide variety of models (Wolpert & Schmidler, 2012). A second problem is that the samples used for HME are obtained from the posterior distribution only. This leads to the opposite behavior as for AME: because the contribution of the prior to the LME might not be appropriately accounted for, the HME tends to overestimate the model evidence, a behavior that can be difficult to diagnose (Lartillot & Philippe, 2006). Several improvements of the HME have been proposed to account for this shortcoming (for example, Raftery et al., 2007).

Implementation

Since TI requires samples from both the prior and the posterior distribution, which correspond to the power posteriors with $\beta = 0$ and $\beta = 1$, respectively, the samples acquired for TI can be used for computing the other sampling-based estimators, AME and HME. In our comparisons throughout this paper, we have used this technique to ensure that any observed differences between estimators are not simply due to differences in the implementation of the samplers.

S7 Connectivity parameters of the synthetic models

The connectivity parameters of the synthetic models used here are shown below.

Model 1

Model one did not include any bilinear or non-linear terms.

$$A = \begin{pmatrix} -0.5 & 0 & 0 \\ 0 & -0.5 & 0 \\ 0 & 0 & -0.5 \end{pmatrix}, \quad C = \begin{pmatrix} 1 & 0 \\ 0 & 1 \\ 1 & 1 \end{pmatrix}.$$

Model 2

Models 2 to 5 used the same A and C matrices. In addition, models 2 to 4 included one bilinear term (B matrices), and model 5 included a nonlinear term (D matrices).

$$A = \begin{pmatrix} -0.5 & 0 & -0.25 \\ 0 & -0.5 & -0.25 \\ 0.5 & 0.5 & -0.5 \end{pmatrix}, \quad C = \begin{pmatrix} 1 & 0 \\ 0 & 1 \\ 0 & 0 \end{pmatrix},$$

$$B_1 = \begin{pmatrix} 0 & 0 & 0 \\ 0 & 0 & 0 \\ 0 & 3 & 0 \end{pmatrix}, \quad B_2 = \begin{pmatrix} 0 & 0 & 0 \\ 0 & 0 & 0 \\ 0 & 0 & 0 \end{pmatrix}.$$

Model 3

Because model 3 shared the same A and C matrix with model 2, we only display the B matrices.

$$B_1 = \begin{pmatrix} 0 & 0 & 0 \\ 0 & 0 & 0 \\ 0 & 0 & 0 \end{pmatrix}, \quad B_2 = \begin{pmatrix} 0 & 0 & 0 \\ 0 & 0 & 0 \\ 3 & 0 & 0 \end{pmatrix}.$$

Model 4

Again, only the B matrices differed between models 2, 3, and 4.

$$B_1 = \begin{pmatrix} 0 & 0 & 0 \\ 0 & 0 & 0 \\ 0 & 0 & 0 \end{pmatrix}, \quad B_2 = \begin{pmatrix} 0 & 0 & 0 \\ 0 & 0 & 0 \\ 0 & 0 & -2 \end{pmatrix}.$$

Model 5

Model 5 included no bilinear term but included one non-linear term.

$$D_1 = \begin{pmatrix} 0 & 0 & 0 \\ 0 & 0 & 0 \\ 0 & 0 & 0 \end{pmatrix}, \quad D_2 = \begin{pmatrix} 0 & 0 & 0 \\ 0 & 0 & 0 \\ 1 & 0 & 0 \end{pmatrix}, \quad D_3 = \begin{pmatrix} 0 & 0 & 0 \\ 0 & 0 & 0 \\ 0 & 0 & 0 \end{pmatrix}.$$

The exact data structures can be downloaded from the ETH research collection (ETH Zurich, 2020).

S8 Scaling of BOLD signals

In the SPM version used here (5236), BOLD signals y are rescaled with respect to their ℓ_∞ norm, such that

$$||y||_\infty = 4. \tag{A.1}$$

In DCM, the observation equation (see Eq. 4) can be written as

$$y = g(h, \theta_g) + X_0\beta + \varepsilon \qquad (A.2)$$

where X_0 represents confounding factors. This matrix usually consists of cosine functions that account for baseline effects and low frequency components and can be imagined as implementing a model of structured noise (scanner-related fluctuations in signal intensity) that is distinct from the model's residuals. We assume N observations such that data from a region is $y[t],\ t = 0, \dots, N-1$, and the components of $X_0 = [x_K, \dots, x_{M-1}]^T$, $K > 0$ are

$$x_k[t] = \cos(\frac{2\pi kt}{N}). \qquad (A.3)$$

In this case, $X_0^T X_0$ is a diagonal matrix as all base functions are orthogonal. The diagonal elements are given by

$$\sum_{n=0}^{N-1} \cos\left(\frac{2\pi\omega n}{N}\right)^2 = \frac{N}{2}, \qquad (A.4)$$

Thus,

$$X_0^T X_0 = \frac{N}{2} I. \qquad (A.5)$$

The posterior variance of the regressors conditioned on the predictions from DCM, the variance of the error σ_c^2, and the prior variance σ_0, is

$$(\sigma_c^{-2} X^T X + \sigma_0^{-2} I)^{-1} = \left(\frac{\sigma_c^{-2} N}{2} I + \sigma_0^{-2} I\right)^{-1}, \qquad (A.6)$$

$$= \left(\frac{N}{2\sigma_c^2} + \frac{1}{\sigma_0^2}\right)^{-1} I. \qquad (A.7)$$

To derive the prior variance of the signal predicted by $X_0\beta$, we note that for the predicted signal y:

$$E[y[t]^2] = E\left[\left(\sum_{\omega=K}^{M-1} \beta_\omega \cos\frac{2\pi t\omega}{N}\right)^2\right], \qquad (A.8)$$

$$= E\left[\sum_{\omega,k=K}^{M-1} \beta_\omega \beta_k \cos\frac{2\pi t\omega}{N} \cos\frac{2\pi tk}{N}\right]. \qquad (A.9)$$

Because the coefficients are assumed to be uncorrelated and to have zero mean, it follows that

$$= \sum_{\omega=K}^{M-1} Var(\beta_\omega) \cos^2\left(\frac{2\pi t\omega}{N}\right), \quad (A.10)$$

$$= \sigma_0^2 \left(\sum_{\omega=0}^{M-1} \cos^2\left(\frac{2\pi t\omega}{N}\right) - \sum_{\omega=0}^{K-1} \cos^2\left(\frac{2\pi t\omega}{N}\right) \right). \quad (A.11)$$

Assuming that $2Mt/N$ is an integer, it follows that

$$= \sigma_0^2 \left(\frac{M}{2} - \sum_{\omega=0}^{K-1} \cos^2\left(\frac{2\pi t\omega}{N}\right) \right). \quad (A.12)$$

It follows that

$$\frac{\sigma_0^2 (M-K)}{2} \leq E[y[t]^2] = Var(y[t]) \leq \frac{\sigma_0^2 M}{2}. \quad (A.13)$$

This constitutes an approximation to the prior variance of the signal. Although in the SPM implementation of DCM used here, σ_0^2 is set to 10^8, here we use a more pragmatic value $\sigma_0 = ||y||_\infty = 4$. From Eq. A.12, it can be seen that this constitutes a more conservative prior variance than the SPM implementation, but still liberal enough to a priori easily account for the totality of the variance in the data.

S9 Derivation of variational negative free energy under the Laplace approximation

The expression for the variational negative free energy can be derived by noting that Eq. 34 in the main text can be written as an energy term plus an entropy term

$$-F_{VB} = \mathrm{E}[\ln p(y,\theta)]_{q(\theta)} - \mathrm{E}[\ln q(\theta)]_{q(\theta)}. \quad (A.14)$$

For simplicity, in the rest of this section, we collapse parameters Θ and hyperparameters Λ into a d-dimensional vector θ, assuming that a maximum has been obtained. Also, we assume that all densities are conditioned on model m, and make this assumption implicit. Moreover, we assume that the prior distribution of parameters θ is a Gaussian distribution centered at θ_0 with covariance Π_0^{-1}.

According to the Laplace approximation, $q(\theta)$ is a Gaussian distribution with mean $\theta^* = \arg\max_{\theta} p(y,\theta)$ and variance

$$\Pi = -\frac{\partial^2 \ln p(y,\theta)}{\partial\theta^2} = \Pi_0 - \frac{\partial^2 \ln p(y|\theta)}{\partial\theta^2}. \qquad (\mathrm{A.15})$$

We denote the negative Hessian of the likelihood or observed Fisher information in the following as Π_L.

The energy term in Eq. A. 14 is approximated using the Laplace method, which yields

$$E[\ln p(y,\theta)]_{q(\theta)} \approx \ln p(y,\theta^*) - \frac{1}{2}\mathrm{E}[(\theta^* - \theta)'\Pi(\theta^* - \theta)]_{q(\theta)}, \qquad (\mathrm{A.16})$$

$$= \ln p(y,\theta^*) - \frac{1}{2} tr\big(\Pi \mathrm{E}[(\theta^* - \theta)(\theta^* - \theta)']_{q(\theta)}\big), \qquad (\mathrm{A.17})$$

$$= \ln p\,(y,\theta^*) - \frac{1}{2} tr(\Pi\Pi^{-1}) = \ln p(y,\theta^*) - \frac{1}{2}d. \qquad (\mathrm{A.18})$$

where tr denotes the trace operator.

The last term in Eq. A. 14 is the entropy of a Gaussian distribution, which is given by:

$$-E[\ln q(\theta)]_{q(\theta)} = \frac{1}{2}(d\ln 2\pi + d - \ln|\Pi|). \qquad (\mathrm{A.19})$$

where Π is the precision of q.

Plugging Eqs. A. 18 and A. 19 into Eq. A. 14, the variational free energy is given by

$$-F_{VB} = \ln p(y,\theta^*) + \frac{1}{2}(d\ln 2\pi - \ln|\Pi|). \qquad (\mathrm{A.20})$$

The first term on the right of Eq. A. 20 can be expanded to obtain the full expression:

$$\ln p(y,\theta^*) = \ln p(y|\theta^*) + \ln p(\theta^*), \qquad (\mathrm{A.21})$$

$$= \ln p(y|\theta^*) - \frac{1}{2}d\ln 2\pi + \frac{1}{2}\ln|\Pi_0| - \frac{1}{2}(\theta^* - \theta_0)'\Pi_0(\theta^* - \theta_0). \qquad (\mathrm{A.22})$$

where θ_0 and Π_0 are the mean and precision of the prior density, respectively. By inserting Eq. **Error! Reference source not found.** into Eq. A. 20, the scheme proposed by Friston et al. (2007) can be written as:

$$-F_{VB} = \ln p(y|\theta^*) + \frac{1}{2}\ln\frac{|\Pi_0|}{|\Pi|} - \frac{1}{2}(\theta^* - \theta_0)'\Pi_0(\theta^* - \theta_0). \qquad (\mathrm{A.23})$$

Although VBL is typically orders of magnitude faster than MCMC sampling, it exhibits several limitations: it is susceptible to (i) local extrema, (ii) violations of the distributional assumptions imposed on the posterior, (iii) violations of the conditional independence assumptions of the mean field approximation (see Daunizeau, David, & Stephan, 2011 for discussion), and (iv) it is only defined when the Hessian in Eq. A. 15 is not singular.

Returning to our theme of connecting TI to VBL, one can write the variational negative free energy in terms of an approximate accuracy and complexity term (Eq. **Error! Reference source not found.**). One observes that the accuracy term can be computed as

$$-F_{VB} + KL\big(q(\theta)||p(\theta)\big) = A_{VB}. \qquad (A.24)$$

Given a Gaussian prior and posterior, the KL divergence has the following analytical form:

$$KL\big(q(\theta)||p(\theta)\big) = \frac{1}{2}\left[\ln\frac{|\Pi|}{|\Pi_0|} + tr(\Pi_0\Pi^{-1}) - d + (\theta^* - \theta_0)'\Pi_0(\theta^* - \theta_0)\right]. \qquad (A.25)$$

Replacing terms, we obtain

$$A = E[\ln p(y|\theta)]_{q(\theta)}, \qquad (A.26)$$

$$\approx \mathrm{A_{VB}} = \ln p(y|\theta^*) + \frac{tr(\Pi_0\Pi^{-1})}{2} - \frac{d}{2}. \qquad (A.27)$$

A more familiar expression for the accuracy can be derived by noting that the posterior covariance can be written as the sum of the negative Hessian of the likelihood plus the prior covariance, such that

$$\mathrm{A_{VB}} = \ln p(y|\theta^*) + \frac{1}{2}tr\left(\frac{\Pi_0 + \Pi_L - \Pi_L}{\Pi_0 + \Pi_L}\right) - \frac{d}{2}, \qquad (A.28)$$

$$= \ln p(y|\theta^*) - \frac{1}{2}tr\left(\frac{\Pi_L}{\Pi_0 + \Pi_L}\right), \qquad (A.29)$$

$$\mathbb{p} = tr\left(\frac{\Pi_L}{\Pi_0 + \Pi_L}\right). \qquad (A.30)$$

$\mathbb{p}$ is the effective number of parameters proposed by Moody (1991) Eq. 18 and see Spiegelhalter, Best, Carlin, and van der Linde (2002) Eq. 15 and is commonly used for model selection.

S10 Predicted fMRI time series for the attention to motion dataset

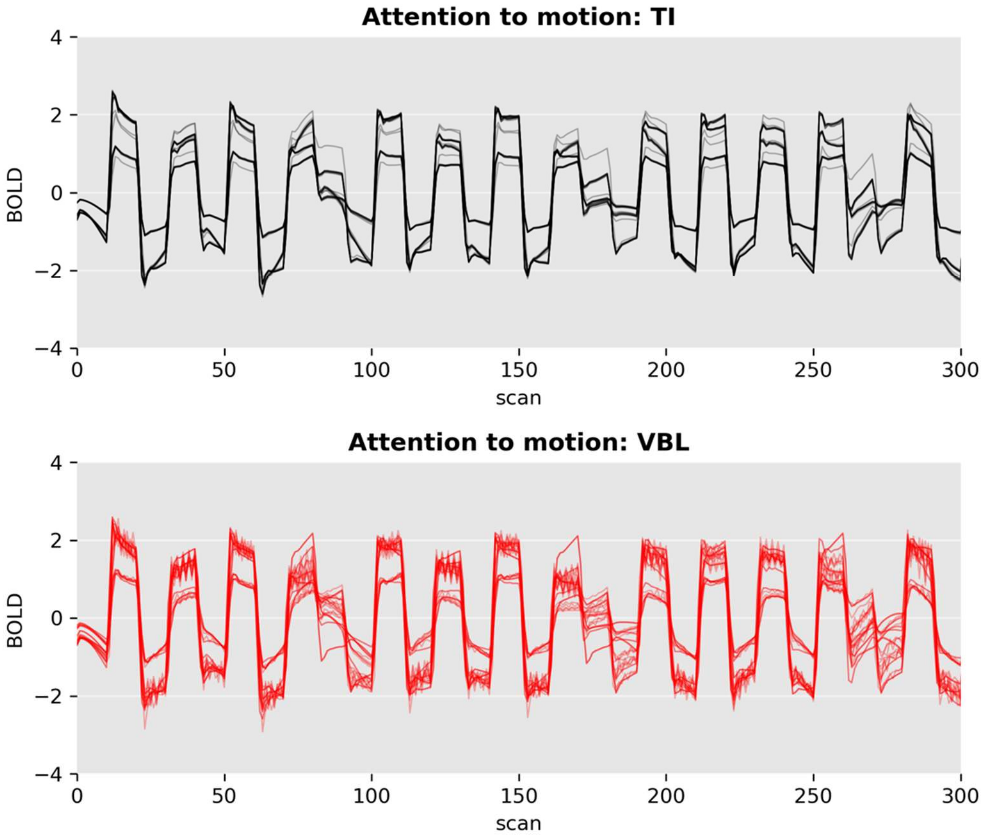

Figure S1. Comparison of 10 predicted BOLD signal trajectories (for the MAP estimate) of model m_4 between TI and VBL for the "attention to motion" dataset from Buchel (1997). In order to obtain an unbiased impression of the variability, the predicted BOLD responses are plotted in full (i.e., including estimated confounds; compare Eq. 4). Both estimates are qualitatively similar, but VBL fits display higher variability.

S11 Final step in the derivation of the fundamental TI equation

Applying the chain rule of differentiation to the logarithm of a positive-valued function, we have the following relation:

$$\frac{d}{d\beta}\ln f(\beta) = \frac{1}{f(\beta)}\frac{d}{d\beta}f(\beta)$$

In the main text section Thermodynamic Integration and the origin of free energy, we have shown that the log-model evidence is given by the expression (Eq. 22 main text):

$$\ln p(y|m) = \int_{\beta=0}^{\beta=1} \frac{d}{d\beta}\ln \int p(y|\theta,m)^{\beta} p(\theta|m)\, d\theta\, d\beta$$

Applying the above relation with $f(\beta) = \int p(y|\theta, m)^\beta p(\theta|m)\, d\theta = Z_\beta$, we have

$$\frac{d}{d\beta}\ln \int p(y|\theta, m)^\beta p(\theta|m)\, d\theta = \frac{\frac{d}{d\beta}\int p(y|\theta, m)^\beta p(\theta|m)\, d\theta}{\int p(y|\theta, m)^\beta p(\theta|m)\, d\theta}$$

$$= \frac{1}{Z_\beta}\int \frac{d}{d\beta} p(y|\theta, m)^\beta p(\theta|m)\, d\theta$$

$$= \frac{1}{Z_\beta}\int p(y|\theta, m)^\beta p(\theta|m) \ln p(y|\theta, m)\, d\theta$$

$$= \int \frac{p(y|\theta, m)^\beta p(\theta|m)}{Z_\beta} \ln p(y|\theta, m)\, d\theta.$$

Note that the last line above is the integrand in Eq. 23 in the main text. Also note that in the second line above, we have exchanged the derivative with respect to β with the integration over θ and in the third line, we have used the derivative of an exponential function:

$$\frac{d}{d\beta} a^\beta = a^\beta \ln a.$$

References

Aponte, E. A., Raman, S., Sengupta, B., Penny, W., Stephan, K. E., & Heinzle, J. (2016). mpdcm: A toolbox for massively parallel dynamic causal modeling. *Journal of Neuroscience Methods, 257*, 7-16. doi:http://dx.doi.org/10.1016/j.jneumeth.2015.09.009

Blundell, S. J., & Blundell, K. M. (2009). *Concepts in Thermal Physics*. Oxford: Oxford University Press, Incorporated.

Brooks, S., Gelman, A., Jones, G., & Meng, X. L. (2011). *Handbook of Markov chain Monte Carlo* (S. Brooks, A. Gelman, G. Jones, & X. L. Meng Eds.). New York: Chapman & Hall.

Buchel, C. (1997). Modulation of connectivity in visual pathways by attention: cortical interactions evaluated with structural equation modelling and fMRI. *Cerebral cortex (New York, N.Y. 1991), 7*(8), 768-778. doi:10.1093/cercor/7.8.768

Buxton, R. B., Wong, E. C., & Frank, L. R. (1998). Dynamics of blood flow and oxygenation changes during brain activation: The balloon model. *Magnetic Resonance in Medicine, 39*(6), 855-864. doi:10.1002/mrm.1910390602

Calderhead, B., & Girolami, M. (2009). Estimating Bayes factors via thermodynamic integration and population MCMC. *COMPUTATIONAL STATISTICS & DATA ANALYSIS, 53*, 4028-4045. doi:10.1016/j.csda.2009.07.025

Daunizeau, J., David, O., & Stephan, K. E. (2011). Dynamic causal modelling: A critical review of the biophysical and statistical foundations. *NeuroImage, 58*(2), 312-322. doi:http://dx.doi.org/10.1016/j.neuroimage.2009.11.062

ETH Zurich. (2020). ETH Research Collection. Retrieved from https://www.research-collection.ethz.ch/bitstream/handle/20.500.11850/301664/simulation_dcms.zip

Friston, K. J. (2002). Bayesian Estimation of Dynamical Systems: An Application to fMRI. *NeuroImage, 16*(2), 513-530. doi:http://dx.doi.org/10.1006/nimg.2001.1044

Friston, K. J., & Dolan, R. J. (2010). Computational and dynamic models in neuroimaging. *NeuroImage (Orlando, Fla.), 52*(3), 752-765. doi:10.1016/j.neuroimage.2009.12.068

Friston, K. J., Harrison, L., & Penny, W. (2003). Dynamic causal modelling. *NeuroImage, 19*(4), 1273-1302. doi:10.1016/S1053-8119(03)00202-7

Friston, K. J., Litvak, V., Oswal, A., Razi, A., Stephan, K. E., van Wijk, B. C. M., . . . Zeidman, P. (2016). Bayesian model reduction and empirical Bayes for group (DCM) studies. *NeuroImage, 128*(Supplement C), 413-431. doi:https://doi.org/10.1016/j.neuroimage.2015.11.015

Friston, K. J., Mattout, J., Trujillo-Barreto, N., Ashburner, J., & Penny, W. (2007). Variational free energy and the Laplace approximation. *NeuroImage, 34*(1), 220-234. doi:http://dx.doi.org/10.1016/j.neuroimage.2006.08.035

Gelman, A., & Rubin, D. B. (1992). Inference from Iterative Simulation Using Multiple Sequences. *Statistical Science, 7*(4), 457-472. doi:10.1214/ss/1177011136

Henson, R. N., Mattout, J., Phillips, C., & Friston, K. J. (2009). Selecting forward models for MEG source-reconstruction using model-evidence. *NeuroImage (Orlando, Fla.), 46*(1), 168-176. doi:10.1016/j.neuroimage.2009.01.062

Jaynes, E. T. (1957). Information Theory and Statistical Mechanics. *Physical Review, 106*(4), 620-630. doi:10.1103/physrev.106.620

Kass, R. E., & Raftery, A. E. (1995). Bayes Factors. *Journal of the American Statistical Association, 90*(430), 773-795. doi:10.1080/01621459.1995.10476572

Koller, D. (2009). *Probabilistic graphical models : principles and techniques*. Cambridge, Mass: MIT Press.

Lartillot, N., & Philippe, H. (2006). Computing Bayes Factors Using Thermodynamic Integration. *Systematic Biology, 55*(2), 195-207. doi:10.1080/10635150500433722

Lomakina, E. I., Paliwal, S., Diaconescu, A. O., Brodersen, K. H., Aponte, E. A., Buhmann, J. M., & Stephan, K. E. (2015). Inversion of hierarchical Bayesian models using Gaussian processes. *NeuroImage, 118*, 133-145. doi:http://dx.doi.org/10.1016/j.neuroimage.2015.05.084

MacKay, D. J. C. (2004). *Information Theory, Inference, and Learning Algorithms* (Repr. with corr. ed.). Cambridge: Univ. Press.

McDowell, J. E., Dyckman, K. A., Austin, B. P., & Clementz, B. A. (2008). Neurophysiology and neuroanatomy of reflexive and volitional saccades: Evidence from studies of humans. *Brain and cognition, 68*(3), 255-270. doi:10.1016/j.bandc.2008.08.016

Moody, J. E. (1991). *The effective number of parameters: an analysis of generalization and regularization in nonlinear learning systems*. Paper presented at the Proceedings of the 4th International Conference on Neural Information Processing Systems, Denver, Colorado.

Penny, W., Stephan, K. E., Daunizeau, J., Rosa, M. J., Friston, K. J., Schofield, T. M., & Leff, A. P. (2010). Comparing Families of Dynamic Causal Models. *PLOS Computational Biology, 6*(3), e1000709. doi:10.1371/journal.pcbi.1000709

Raftery, A., Newton, M., Satagopan, J., & Krivitsky, P. (2007). Estimating the Integrated Likelihood via Posterior Simulation Using the Harmonic Mean Identity. In J. M. Bernardo, M. J. Bayarri, J. O. Berger, A. P. Dawid, D. Heckerman, A. F. M. Smith, & M. West (Eds.), *Bayesian Statistics 8* (pp. 1-45). Oxford: Oxford University Press.

Raman, S., Deserno, L., Schlagenhauf, F., & Stephan, K. E. (2016). A hierarchical model for integrating unsupervised generative embedding and empirical Bayes. *Journal of Neuroscience Methods, 269*, 6-20. doi:http://dx.doi.org/10.1016/j.jneumeth.2016.04.022

Rigoux, L., Stephan, K. E., Friston, K. J., & Daunizeau, J. (2014). Bayesian model selection for group studies — Revisited. *NeuroImage (Orlando, Fla.), 84*, 971-985. doi:10.1016/j.neuroimage.2013.08.065

Robert, C. P., & Casella, G. (2010). *Monte Carlo statistical methods* (2nd ed. ed.): New York : Springer.

Schwarz, G. (1978). Estimating the Dimension of a Model. *The Annals of statistics, 6*(2), 461-464. doi:10.1214/aos/1176344136

Spiegelhalter, D. J., Best, N. G., Carlin, B. P., & van der Linde, A. (2002). Bayesian measures of model complexity and fit. *Journal of the Royal Statistical Society. Series B, Statistical methodology, 64*(4), 583-639. doi:10.1111/1467-9868.00353

Stephan, Klaas E., Iglesias, S., Heinzle, J., & Diaconescu, Andreea O. (2015). Translational Perspectives for Computational Neuroimaging. *Neuron, 87*(4), 716-732. doi:http://dx.doi.org/10.1016/j.neuron.2015.07.008

Stephan, K. E., Kasper, L., Harrison, L. M., Daunizeau, J., den Ouden, H. E. M., Breakspear, M., & Friston, K. J. (2008). Nonlinear dynamic causal models for fMRI. *NeuroImage, 42*(2), 649-662. doi:http://dx.doi.org/10.1016/j.neuroimage.2008.04.262

Stephan, K. E., Penny, W., Daunizeau, J., Moran, R. J., & Friston, K. J. (2009). Bayesian model selection for group studies. *NeuroImage, 46*(4), 1004-1017. doi:http://dx.doi.org/10.1016/j.neuroimage.2009.03.025

Stephan, K. E., Schlagenhauf, F., Huys, Q. J. M., Raman, S., Aponte, E. A., Brodersen, K. H., . . . Heinz, A. (2017). Computational neuroimaging strategies for single patient predictions. *NeuroImage, 145, Part B*, 180-199. doi:https://doi.org/10.1016/j.neuroimage.2016.06.038

Stephan, K. E., Weiskopf, N., Drysdale, P. M., Robinson, P. A., & Friston, K. J. (2007). Comparing hemodynamic models with DCM. *NeuroImage, 38*(3), 387-401. doi:http://dx.doi.org/10.1016/j.neuroimage.2007.07.040

Swendsen, R. H., & Wang, J.-S. (1986). Replica Monte Carlo Simulation of Spin-Glasses. *Physical Review Letters, 57*(21), 2607-2609. doi:10.1103/physrevlett.57.2607

Trujillo-Barreto, N. J., Aubert-Vázquez, E., & Valdés-Sosa, P. A. (2004). Bayesian model averaging in EEG/MEG imaging. *NeuroImage (Orlando, Fla.), 21*(4), 1300-1319. doi:10.1016/j.neuroimage.2003.11.008

Vyshemirsky, V., & Girolami, M. A. (2008). Bayesian ranking of biochemical system models. *Bioinformatics, 24*(6), 833-839. doi:10.1093/bioinformatics/btm607

Wipf, D., & Nagarajan, S. (2009). A unified Bayesian framework for MEG/EEG source imaging. *NeuroImage, 44*(3), 947-966. doi:https://doi.org/10.1016/j.neuroimage.2008.02.059

Wolpert, R. L., & Schmidler, S. C. (2012). α-STABLE LIMIT LAWS FOR HARMONIC MEAN ESTIMATORS OF MARGINAL LIKELIHOODS. *Statistica Sinica, 22*(3), 1233-1251. doi:10.5705/ss.2010.221

Yao, Y., Raman, S. S., Schiek, M., Leff, A., Frässle, S., & Stephan, K. E. (2018). Variational Bayesian inversion for hierarchical unsupervised generative embedding (HUGE). *NeuroImage, 179*, 604-619. doi:https://doi.org/10.1016/j.neuroimage.2018.06.073
